# Unravelling genetic architecture of circulatory amino acid levels, and their effect on risk of complex disorders

**DOI:** 10.1093/nargab/lqae046

**Published:** 2024-05-06

**Authors:** Leila Abar, Verena Zuber, Georg W Otto, Ioanna Tzoulaki, Abbas Dehghan

**Affiliations:** Department of Epidemiology & Biostatistics, School of Public Health, Faculty of Medicine, Imperial College London, St Mary’s Campus, Norfolk Place, London W2 1PG, UK; Department of Epidemiology & Biostatistics, School of Public Health, Faculty of Medicine, Imperial College London, St Mary’s Campus, Norfolk Place, London W2 1PG, UK; Department of Epidemiology & Biostatistics, School of Public Health, Faculty of Medicine, Imperial College London, St Mary’s Campus, Norfolk Place, London W2 1PG, UK; Department of Epidemiology & Biostatistics, School of Public Health, Faculty of Medicine, Imperial College London, St Mary’s Campus, Norfolk Place, London W2 1PG, UK; Centre for Systems Biology, Biomedical Research Foundation Academy of Athens, 115 27 Athens, Greece; BHF Centre of Excellence, School of Public Health, Imperial College London, London W2 1PG, UK; UK Dementia Research Institute, Imperial College London, London W12 0BZ, UK; Department of Epidemiology & Biostatistics, School of Public Health, Faculty of Medicine, Imperial College London, St Mary’s Campus, Norfolk Place, London W2 1PG, UK; BHF Centre of Excellence, School of Public Health, Imperial College London, London W2 1PG, UK; UK Dementia Research Institute, Imperial College London, London W12 0BZ, UK

## Abstract

Variations in serum amino acid levels are linked to a multitude of complex disorders. We report the largest genome-wide association study (GWAS) on nine serum amino acids in the UK Biobank participants (117 944, European descent). We identified 34 genomic loci for circulatory levels of alanine, 48 loci for glutamine, 44 loci for glycine, 16 loci for histidine, 11 loci for isoleucine, 19 loci for leucine, 9 loci for phenylalanine, 32 loci for tyrosine and 20 loci for valine. Our gene-based analysis mapped 46–293 genes associated with serum amino acids, including *MIP*, *GLS2*, *SLC* gene family, *GCKR*, *LMO1*, *CPS1* and *COBLL1*.The gene–property analysis across 30 tissues highlighted enriched expression of the identified genes in liver tissues for all studied amino acids, except for isoleucine and valine, in muscle tissues for serum alanine and glycine, in adrenal gland tissues for serum isoleucine and leucine, and in pancreatic tissues for serum phenylalanine. Mendelian randomization (MR) phenome-wide association study analysis and subsequent two-sample MR analysis provided evidence that every standard deviation increase in valine is associated with 35% higher risk of type 2 diabetes and elevated levels of serum alanine and branched-chain amino acids with higher levels of total cholesterol, triglyceride and low-density lipoprotein, and lower levels of high-density lipoprotein. In contrast to reports by observational studies, MR analysis did not support a causal association between studied amino acids and coronary artery disease, Alzheimer’s disease, breast cancer or prostate cancer. In conclusion, we explored the genetic architecture of serum amino acids and provided evidence supporting a causal role of amino acids in cardiometabolic health.

## Introduction

Circulating levels of amino acids are associated with the development of a variety of diseases, including type 2 diabetes (T2D) (1–4), cardiovascular disease (5–7), inflammatory disease (8,9), non-alcoholic fatty liver disease (10) and Alzheimer’s disease (AD) (11,12), and further with several cardiometabolic risk factors, including insulin resistance, blood pressure and lipid profile (4,7,13). In addition, serum amino acid levels are suggested to be a useful marker for disease status in patients with head and neck cancer (14) as well as for plasma-free amino acid (PFAA) profile, which serves as one of the biological markers for cancer patients (15,16). Nonetheless, most of these observed associations originated from observational studies, and can be influenced by biases and confounding factors. Furthermore, observational studies cannot ascertain causality or determine the direction of the effect. Therefore, identification of the genetic variants associated with the circulating serum amino acid concentrations can be utilized to investigate their impact on chronic conditions. The genetic markers are inherited randomly during conception and thus could be used as a proxy to the exposures in Mendelian randomization (MR) analysis to prevent the biases afflicted in traditional epidemiological studies (17). Genome-wide association studies (GWAS) unravel the genetic determinants of serum amino acids, which, on one hand, could elucidate the role of serum amino acid levels in the pathophysiology of complex disorders and, on the other hand, could be used to conduct MR studies.

Here, we present large-scale GWAS on nine serum levels of amino acids [alanine, isoleucine, leucine and valine (branched-chain amino acids, BCAAs), glycine, glutamine, histidine, phenylalanine and tyrosine] using data from the UK Biobank (UKB) study participants to identify genetic determinants of serum amino acid levels and a post-GWAS analysis to explore the related biological pathways. Moreover, we utilize our GWAS findings and rich phenotypic data from the UKB study to elucidate the potential relationship between serum amino acid concentrations and a broad range of diseases (*n* = 617 clinical diagnoses with >200 cases) through the MR phenome-wide association study (MR-PheWAS) approach and assess the causality. Moreover, we used the largest studies available that use genome-wide association study (GWAS) to look at common complex disorders. These included coronary artery disease (CAD), AD, breast cancer and prostate cancer. We did this to increase the statistical power when looking for associations with these outcomes. This was necessary because the prevalence of these conditions is limited in the UKB.

## Materials and methods

Our research complies with all relevant ethical regulations, as the UKB has ethics approval from the North-West Multi-Centre Research Ethics Committee (11/NW/0382). We utilized data from the UKB under the approved application ID 52569. Additional ethical approval was not required for the present study.

### Study design and study sample

We used data from the UKB study, a large study including over 500 000 participants aged 40–69 years at recruitment from 2006 to 2010. The UKB has used a purpose-designed genotyping array to assay genome-wide genetic data. This array comprised 847 441 genetic polymorphisms. The genotyped data are imputed to 1000 Genomes reference panels (18). High-throughput nuclear magnetic resonance (NMR) metabolic measurements were done using the Nightingale metabolomics platform for a random subset of 117 994 participants at baseline assessment, including measurements of nine amino acids: alanine, glycine, glutamine, histidine, isoleucine, leucine, phenylalanine, tyrosine and valine.

### GWAS on serum amino acid levels in the UKB population

We performed a linear mixed model regression using BOLT-LMM version 2.3 to run GWAS on serum amino acid levels of individuals from European ancestry adjusting for age, sex and the first 10 genetic principal components. The mixed model addresses population stratification and relatedness and therefore minimizes the sample exclusions (19). All serum amino acid levels of individuals from European ancestry of the UKB data were standardized and normalized using rank-based inverse normal transformation. We filtered variants with minor allele frequency <0.01 and Hardy–Weinberg equilibrium *P*-value <0.0001.

### Replication of previously reported SNPs in our UKB GWAS analysis

We sought replication for our lead single-nucleotide polymorphisms (SNPs) in each genomic locus using data from a previously published study by Kettunen *et al.* (20). The study combined data from four large-scale cohort studies, totalling over 24 000 individuals. They employed NMR spectroscopy as the metabolomics platform to measure circulating metabolites. We did a lookup of our lead SNPs in this dataset. The locus was considered replicated if the *P*-value was significant after Bonferroni correction and a concordant effect direction.

### Functional annotation analysis in the UKB population

We applied a range of functional annotation analyses using the FUMA web server (http://fuma.ctglab.nl) (21). All significant SNPs (*P* < 5e−08) and SNPs in linkage disequilibrium (LD) with them (*r*^2^ ≥ 0.6) from the UKB GWAS summary statistics in the previous step were annotated for functional consequences on gene functions, based on Ensemble genes (build 85) using ANNOVAR (21,22). Independent significant SNPs and correlated SNPs were linked to the GWAS catalogue to postulate known associations of the SNPs in the risk loci that were reported previously with several phenotypes (21). Gene mapping was conducted following the acquisition of annotated SNPs. This mapping process employed positional mapping, which relies on the physical distance between the SNPs and the genes (19). For genome-wide gene-based association analysis, FUMA implements MAGMA gene-based analysis (21,23) by mapping the input SNPs to protein-coding genes attained from Ensemble build 92. Same as for gene-based analysis, FUMA implements MAGMA (21) for gene-set enrichment analysis, which is conducted for curated gene sets and gene ontology terms attained from Molecular Signatures Database v5.2. Gene-set enrichment analysis assesses whether genes within the gene set are more strongly associated with any of the phenotypes than other genes.

To test the association between tissue-specific gene expression and serum amino acids, MAGMA gene–property analysis was conducted using FUMA (21). This analysis is based on the computed gene-based *P*-values in the gene-based analysis.

### One-sample MR-PheWAS in the UKB population

To explore the effects of nine serum amino acids on a wide range of diseases, we conducted PheWAS analyses. We extracted the phenotypic data of the UKB participants, including patient hospital records, cancer registry data and death registry data defined as ICD-9/10 codes, which were linked to Hospital Episode Statistics, as well as the genotypic data (48–138 amino acid-associated SNPs identified in our GWAS analysis) of the UKB participants (*n* = 117 944). We selected SNPs with *P*-value <1 × 10^−5^ identified in our UKB GWAS with an *F*-statistic >10 (calculated by regressing serum levels per each SNP) as genetic instruments. Multiallelic and palindromic SNPs were excluded, and the remaining SNPs were clumped with *r*^2^ < 0.001. We conducted a one-sample MR analysis approach using the two-stage least-squares method by regressing each serum amino acid on their genetic instruments using a linear regression model and saved the residual values. Subsequently, we used a logistic regression model to regress the saved residual values on each phecode comprising a wide range of clinical diagnoses in the UKB study (*n* = 617 clinical diagnoses with >200 cases). To avoid overlap with the sample that was used for GWAS, the PheWAS analysis was merely done in the ∼385 000 individuals who were not used for GWAS. In addition, the PheWAS analysis was restricted to participants from European ancestry and a kinship coefficient of >0.088 was used to exclude the related individuals. Our PheWAS analysis was adjusted for age, sex and the first 10 genetic principal components. We used the false discovery rate (FDR) method to account for multiple testing as the Bonferroni procedure is suggested to be too conservative due to the correlation between clinical diagnoses.

### Two-sample MR

To further validate the associations identified through our MR-PheWAS analysis, we employed a two-sample MR analysis on five traits, including diabetes, hypertension, disorders of lipid metabolism, hypercholesterolaemia and hyperlipidaemia. We used the same genetic instruments as we selected for MR-PheWAS; however, for their association with outcomes, we used data from the largest GWAS datasets available for each trait and excluded UKB data. We applied the two-sample MR analysis using the inverse-variance weighted (IVW) method. The estimates were calculated per one standard deviation (SD) increase in each serum amino acid level. To further investigate the robustness of the findings to possible pleiotropy, we used weighted median MR (24) and MR-Egger regression (25) as sensitivity analysis. The weighted median MR method assumes that at least half of the instruments are valid instruments. In MR-Egger regression, the intercept indicates the presence of directional pleiotropy, and the slope of that regression will be a consistent estimate of the causal effect of exposure on the outcome of interest even in the presence of invalid instruments (26). Additionally, the MR-PRESSO method was applied to detect and remove outlying genetic variants that substantially affect the IVW estimates (27).

We further conducted a two-sample MR analysis for traits of a number of complex disorders, including CAD (28), late-onset AD (29), breast cancer (30) and prostate cancer (31), using genetic estimate associations reported in the largest GWAS summary statistics in independent populations (Supplementary Table S1).

### Statistical software

All analyses were conducted using R version 4.0.2. Phenome-wide associations were conducted using the R package ‘PheWAS’, and two-sample MR and multivariable MR were performed using ‘TwoSampleMR’ and ‘MRPRESSO’ R packages.

## Results

**Figure 1.**
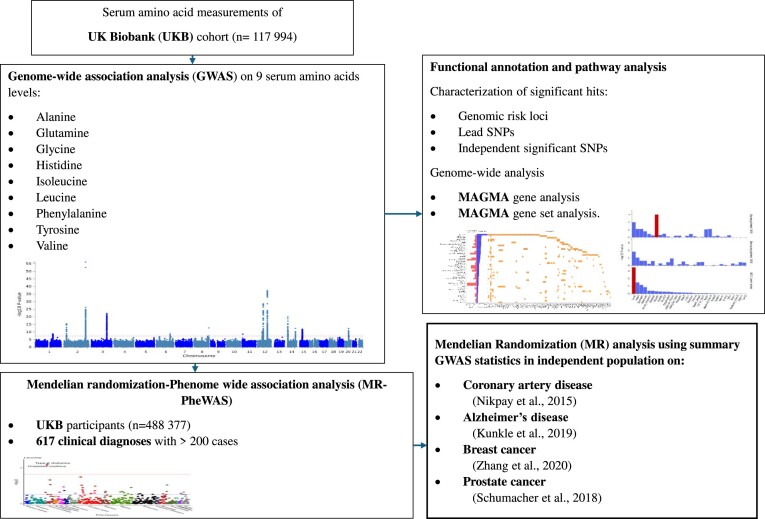
Schematic overview of the study.

The study design is illustrated in Figure 1. We performed a GWAS of nine serum amino acids (alanine, glutamine, glycine, histidine, isoleucine, leucine, phenylalanine, tyrosine and valine) in ∼117 000 population cohort of the UKB study, measured by the Nightingale platform as previously described. Of the 9 933 468 genetic variants included in the analysis, 3225 variants exhibited genome-wide significant associations (*P* < 5 × 10^−8^) with serum alanine, 5619 variants with serum glutamine, 9965 variants with serum glycine, 2008 variants with serum histidine, 2046 variants with serum isoleucine, 1212 variants with serum leucine, 934 variants with serum phenylalanine, 5250 variants with serum tyrosine and 1625 variants with serum valine (Supplementary Tables S2–S11).

### Functional annotation and pathway enrichment

We applied a range of functional annotation analyses to leverage the amino acid GWAS results using FUMA–MAGMA (see Figure 2 for Manhattan plot of GWAS summary statistics). Our FUMA ANNOVAR findings showed that most of the significant SNPs and SNPs in LD with the significant SNPs lie within intronic region followed by intergenic gene regions for all serum amino acids (Supplementary Figures S1–S9).

**Figure 2.**
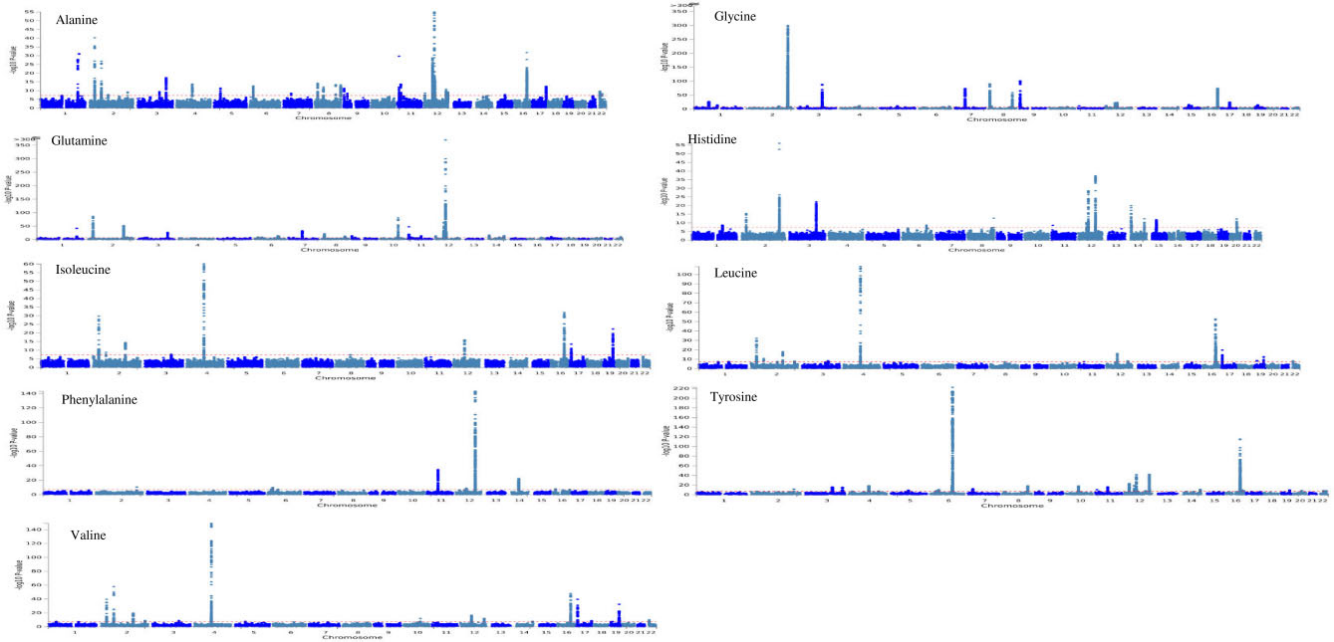
Manhattan plot of GWAS summary statistics for nine serum amino acids.

The findings were mapped to 34 genomic loci for circulatory levels of alanine, 48 loci for glutamine, 44 loci for glycine, 16 loci for histidine, 11 loci for isoleucine, 19 loci for leucine, 9 loci for phenylalanine, 32 loci for tyrosine and 20 loci for valine, by using the FUMA platform (Supplementary Table S2). We identified 20–132 significant lead SNPs (*P* < 5e−8) for each serum amino acid, including 58 for alanine, 131 for glutamine, 132 for glycine, 49 for histidine, 20 for isoleucine, 39 for leucine, 39 for phenylalanine, 109 for tyrosine and 60 for valine (Supplementary Table S2).

Using gene-based analysis implemented in MAGMA, we were able to identify 176 genes for alanine, 293 genes for glutamine, 212 genes for glycine, 95 genes for histidine, 85 genes for isoleucine, 134 genes for leucine, 46 genes for phenylalanine, 152 genes for tyrosine and 145 genes for valine (Supplementary Table S2). In the gene-based analysis, a significant number of genes were found to be shared among all nine serum amino acids. Notably, some of these shared genes include *MIP*, *GLS2*, *SLC* gene family, *GCKR*, *LMO1*, *CPS1* and *COBLL1* (Supplementary Table S12).

Our gene-set MAGMA analysis revealed that prioritized gene sets are involved in the regulation of metabolites (small molecules and protein measures) and PFAA levels (adjusted for 20 other PFAAs), except for serum aromatic amino acids (phenylalanine and tyrosine). Moreover, the pathway enrichment analysis demonstrated the involvement of serum amino acids in the regulation of fasting blood glucose levels, blood lipid profile including triglycerides, cholesterol, high-density lipoprotein (HDL) and low-density lipoprotein (LDL), blood pressure (both systolic and diastolic), as well as mean arterial pressure, coronary heart disease, C-reactive protein levels and many more (Supplementary Figures S10–S18).

**Figure 3.**
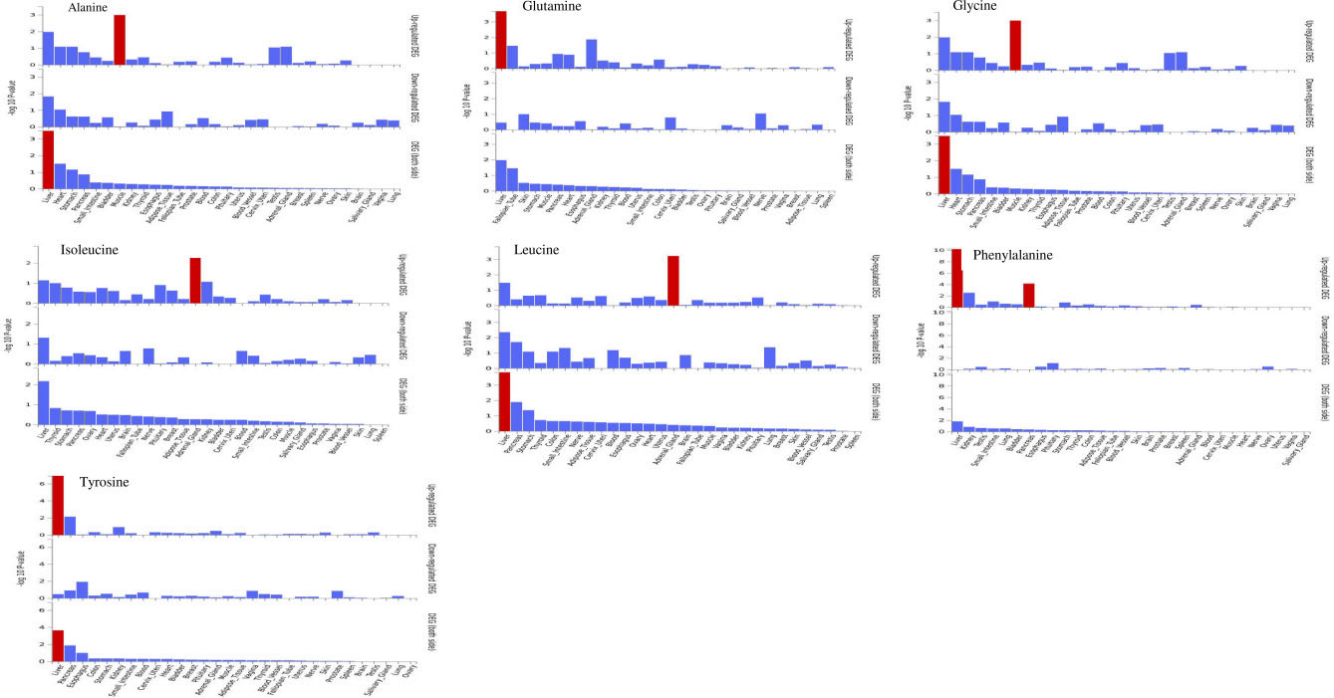
Gene–property analysis across 30 tissues for serum amino acids.

The gene–property analysis across 30 tissues highlighted the expression of most of serum amino acids in liver tissues, except for serum isoleucine and valine, in muscle tissues for serum alanine and glycine, in adrenal gland tissues for serum isoleucine and leucine, and in pancreatic tissues for serum phenylalanine (Figure 3). This analysis did not highlight any further significant associations for the other amino acid levels, including histidine and valine. The total proportion of variance explained (*R*²) for serum amino acids, based on selected instruments, ranged from 1% for isoleucine with a combined *F*-statistic of 24.35 for 60 SNPs to 6% for serum glycine with a combined *F*-statistic of 53.58 for 120 SNPs. The proportion of variance explained for serum BCAAs (isoleucine, leucine and valine) ranged from 1% to 3% with an *F*-statistic of 24–34 (Supplementary Table S13).

### Replication of significant identified loci

We looked up our lead SNPs for associations with serum amino acids in a previously published independent study (*N* = 24 925) (20). We replicated (*P* < 0.01) 4 variants for leucine, 3 variants for phenylalanine, 6 variants for isoleucine and valine, 9 variants for histidine and glycine, 14 variants for tyrosine and 17 variants for alanine (see the ‘Materials and methods’ section and Supplementary Tables S14–S22 for full replication results).

### One-sample MR-PheWAS in the UKB population

**Figure 4.**
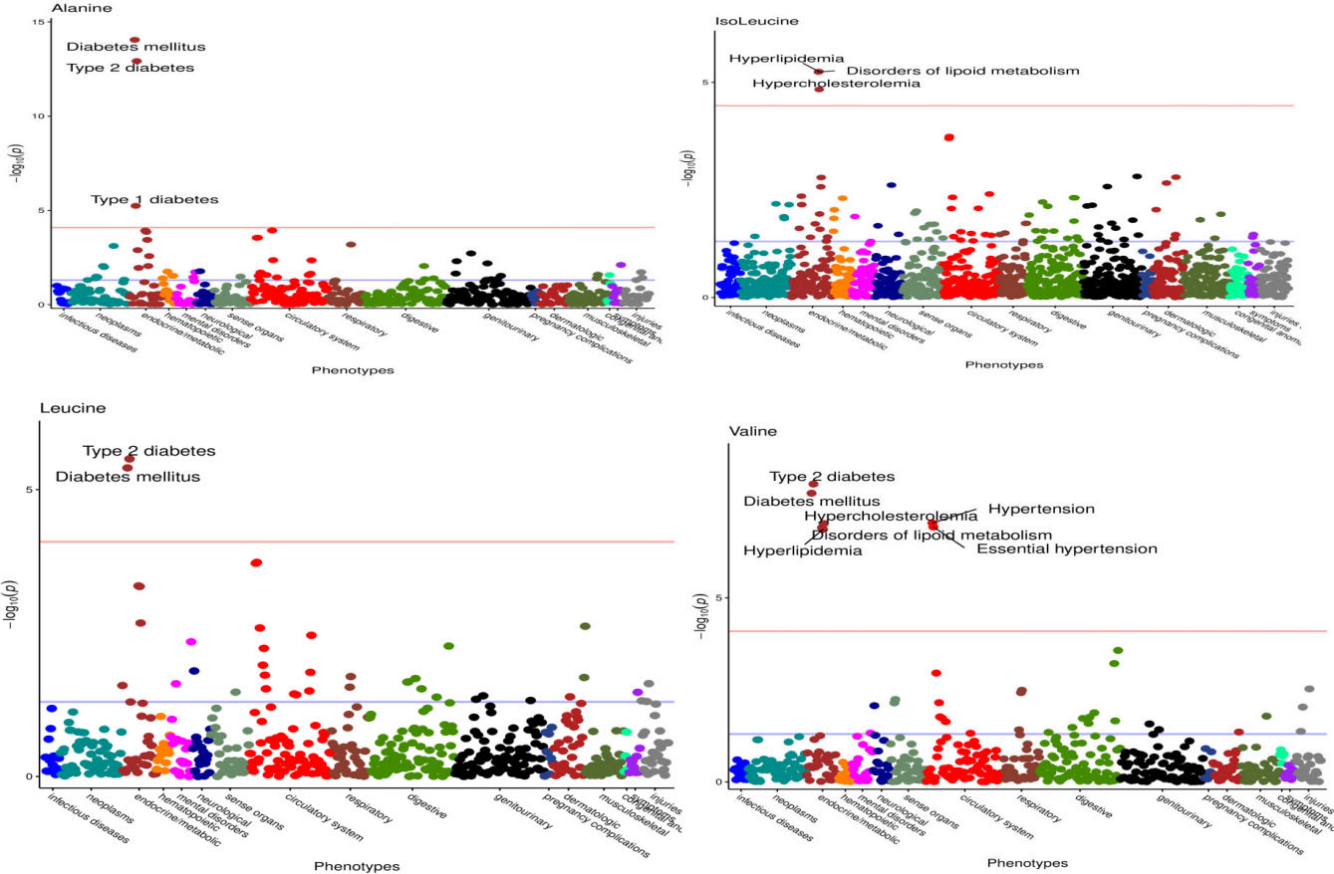
MR-PheWAS Manhattan plot for serum alanine and BCAAs.

We performed PheWAS utilizing genetic variants for all serum amino acids. After clumping (*r*^2^ < 0.001) and excluding the multiallelic SNPs, we had 101 independent (*r*² <0.001) genome-wide significant SNPs (*P* < 5e−08) for serum alanine, 120 for glutamine, 138 for glycine, 62 for histidine, 60 for isoleucine, 67 for leucine, 48 for tyrosine, 110 for phenylalanine and 91 for valine (Supplementary Tables S3–S11). Out of nine serum amino acids, we found clinical diagnoses associated with only four of them, which included alanine and three BCAAs (leucine, isoleucine and valine). Figure 4 shows the Manhattan plot for these analyses. Serum alanine, leucine and valine were significantly associated with the risk of diabetes [odds ratio (OR) = 1.80 per SD and *P*= 1.61e−11 for serum alanine; OR = 1.58 and *P*= 0.003 for serum leucine; OR = 1.62 and *P*= 0.02 for isoleucine] (Supplementary Table S23).

Serum isoleucine and valine were associated with disorders of lipid metabolism (OR = 1.53 per SD and *P*= 0.001 for serum isoleucine; OR = 1.38 per SD and *P*= 3.60e−05 for serum valine), hyperlipidaemia (OR = 1.53 per SD and *P*= 0.001 for serum isoleucine; OR = 1.38 per SD and *P*= 3.60e−05 for serum valine) and hypercholesterolaemia (OR = 1.54 per SD and *P*= 0.001 for serum isoleucine; OR = 1.40 per SD and *P*= 3.60e−05 for serum valine). Serum valine was also significantly associated with hypertension (OR = 1.28 per SD and *P*= 3.60e−05) (Figure 3 and Supplementary Table S23).

No significant association was observed for serum histidine, glycine, glutamine, tyrosine and phenylalanine across phenome.

### Validation of PheWAS results using two-sample MR in the UKB population

**Figure 5.**
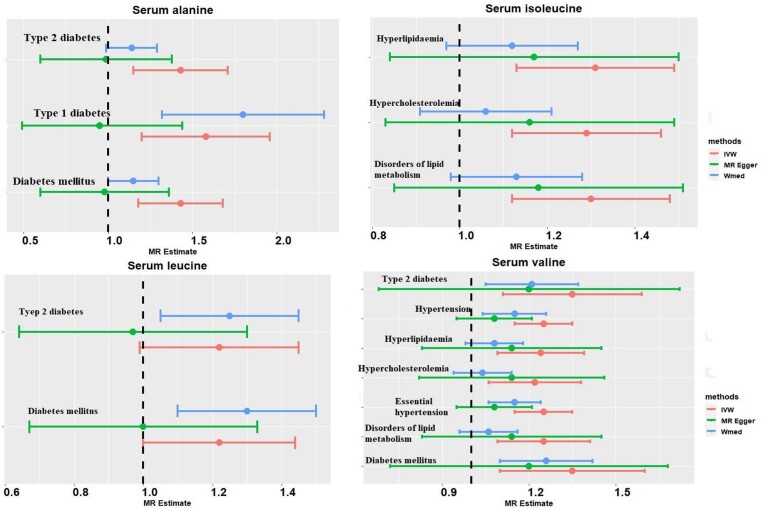
Two-sample MR analysis on studied amino acids and clinical diagnoses using data from the UKB.

We conducted a two-sample MR analysis on findings from PheWAS using data from the UKB. Figure 5 depicts significant associations between genetically predicted serum levels of alanine [OR for IVW = 1.43; 95% confidence interval (CI) = 1.18–1.74] and leucine (OR for IVW = 1.22; 95% CI = 1.02–1.46) and an increased risk of diabetes. Moreover, we found a significant association of serum valine levels with T2D and an increased risk of diabetes (OR for IVW = 1.35; 95% CI = 1.10–1.64) and hypertension (OR for IVW = 1.25; 95% CI = 1.15–1.35), with consistency across both IVW and weighted median estimates. After removing the outliers by the MR-PRESSO method, results were also consistent with the IVW estimate (Supplementary Table S24).

**Figure 6.**
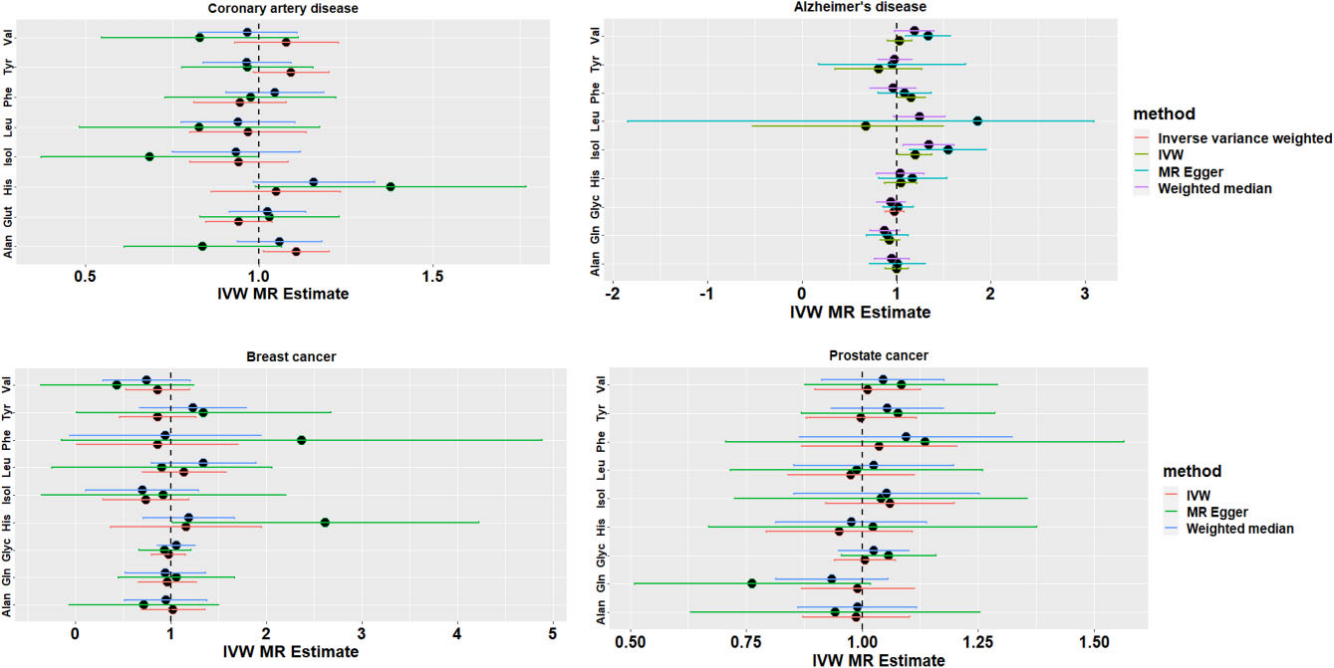
MR results for nine serum amino acids and complex disorders.

We did not find a significant association between genetically elevated levels of any serum amino acids and the risk of CAD (Figure 6 and Supplementary Table S25). Among all amino acids, genetically elevated levels of serum isoleucine were associated with the risk of AD; however, the association did not remain significant after correcting for multiple testing using FDR (Figure 6 and Supplementary Table S26). No association was observed between genetically elevated levels of serum amino acids and risk of breast cancer (Figure 6 and Supplementary Table S27) and prostate cancer (Figure 6 and Supplementary Table S28).

## Discussion

We have conducted the most extensive GWAS of nine serum amino acid levels, involving ∼117 000 participants from the UKB study. Our research significantly enhances the current understanding of the genetic architecture of serum amino acid levels. Furthermore, we provided a systematic evaluation of potential causal associations between serum amino acids and a range of diseases and clinical traits. In our study, we uncovered a spectrum of genomic risk loci, varying in number from 9 for serum phenylalanine to 48 for serum glutamine. We have created a diverse array of genetic variants that can be used as instrumental variables to explore the underlying biological mechanisms and to facilitate further research into potential links between serum amino acids and the risk of complex disorders. These genetic variants accounted for different proportions of variance, ranging from 1–3% for serum BCAAs to 6% for serum glycine and glutamine, respectively.

Our investigation unveiled that genetically predicted higher levels of serum valine were associated with an increased risk of T2D. Furthermore, higher levels of genetically predicted serum alanine and BCAAs were linked to elevated cholesterol, triglyceride and LDL levels, while being associated with lower levels of HDL.

We report a potential causal association between BCAAs and dyslipidaemia, which is supported by evidence linking higher BCAA levels with dyslipidaemia and higher triglyceride levels (5,6). However, the connection between serum BCAAs and LDL cholesterol levels remains unclear, requiring further exploration to disentangle the complexities linking BCAAs, lipid metabolism and cardiovascular disease risk. We further report a potential causal association between serum BCAA levels and diabetes, as supported by previous observational (32,33) and MR studies (2,3). Unlike prior studies, our analysis leverages data from over 117 000 individuals, offering a comprehensive examination of the genetic determinants of serum BCAA levels and their correlation with diabetes.

Our findings are in agreement with observational studies associating higher BCAA levels with cardiometabolic risk factors and CAD (7,34,35), but also expand on these insights by suggesting a possible increase in cardiovascular disease risk with raised isoleucine levels supported by observational evidence (36). Our results are in good agreement with a nested case-control study on BCAAs and risk of breast cancer (37), and may explain why studies on dietary BCAA intake and risk of breast cancer are inconclusive (38,39). Despite these findings, no significant associations were found between increased serum amino acids and the risk of CAD, AD or cancers such as breast cancer or prostate cancer. This could be due to the limited number of cases in the UKB, potentially leading to insufficient statistical power for detecting such associations and highlighting the need for larger studies to further explore these associations.

The association of elevated serum alanine levels with T2D and dyslipidaemia identified in the study is an interesting finding that fits very well with alanine’s role in both glucogenic and proteogenic pathways, especially during conditions of physiological stress such as fasting (40). The breakdown of muscle proteins releases alanine into the bloodstream, which is then utilized by the liver for gluconeogenesis, essential for maintaining blood glucose levels during fasting (40). The gluconeogenic pathway, deeply entangled with lipid metabolism, could potentially influence serum lipid profiles, thus linking alanine with dyslipidaemia. Additionally, alanine might affect the activity of AMP-activated protein kinase, which is key for cellular energy homeostasis, and in turn could enhance fatty acid oxidation and suppress lipid synthesis (41). The resulting increase in gluconeogenic flux may disrupt the energy balance within hepatocytes, leading to upregulation of lipogenesis and elevation of triglyceride levels. The altered hepatic conversion of alanine to pyruvate and subsequent changes in acetyl-CoA levels could further exacerbate these dyslipidaemic profiles (42).

We also found a potential causal link between blood levels of BCAAs and lipid profiles, proposing that BCAAs, such as leucine, could activate the mTOR signalling pathway in a manner distinct from insulin, suggesting that BCAAs may directly affect lipid metabolism and contribute to dyslipidaemia (43,44). Moreover, MAGMA gene-based analysis further identified genes such as GCKR, a highly pleiotropic gene associated with glucose metabolism and fasting plasma glucose levels, and the SLC superfamily, which is involved in the transmembrane transport of essential compounds, including amino acids. These genes are implicated in a variety of conditions, such as dyslipidaemia and neurodegenerative disorders, establishing a genetic link to the observed metabolic alterations (45–50). The gene–property analysis also contributed to understanding the expression patterns of serum amino acids, indicating the significant expression of alanine in muscle tissues and its potential as a biological marker for cardiometabolic health (51–53).

Our MAGMA gene-set analysis further indicated that serum amino acids are integral in pathways regulating serum metabolites, PFAA profiles and fasting glucose levels. These pathways are closely linked with cardiometabolic risk factors such as blood pressure, lipid levels and diabetes. PFAA profiles have been associated with the future risk of diabetes and cardiovascular disease in both non-diabetic and diabetic populations, as well as with Crohn’s disease, underlining their importance in disease prognosis and the potential for targeted therapeutic interventions (54–56).

BCAA levels prior to the onset of T2D are altered, suggesting a role in disease progression. Epidemiological data indicate that changes in BCAA metabolism are associated with cardiometabolic health detriments such as insulin resistance, elevated blood pressure and dyslipidaemia, which are indicators of CAD (7,13,57). Insulin is a key regulator of BCAA catabolism, acting through the BCKD (branched-chain alpha-keto acid dehydrogenase) complex. It is suggested that insulin resistance, common in obesity and T2D, may elevate BCAA levels due to reduced activity of the BCKD enzyme, leading to altered glucose homeostasis and an increased risk of developing T2D (58).

The findings from our study offer significant insights into the clinical implications of serum amino acid levels on cardiometabolic health. The genetic associations identified with serum valine levels suggest a potential causal relationship with the risk of developing T2D, underscoring the potential of monitoring and managing these amino acid levels to prevent or treat T2D. Furthermore, the study establishes a link between elevated serum alanine and BCAAs with dyslipidaemia, characterized by increased cholesterol and triglyceride levels, and decreased HDL. Further research is needed to examine whether serum amino acid profiling could be added as a part of risk assessment for patients predisposed to cardiometabolic disorders, particularly T2D. The study’s implications suggest that dietary modifications or targeted supplementation could be explored as potential strategies to mitigate risk.

### Strengths and limitations

The strength of this investigation includes the agnostic nature of the PheWAS and the large sample size of the UKB study for both GWAS (∼117 000 individuals) and PheWAS analyses (*n* = 323 223). The PheWAS design enabled us to agnostically investigate the association of serum amino acid status with a wide range of phenotypes (617 phenotypes). Finally, we validated the MR-PheWAS results using GWAS in independent samples as well as summary statistics from published GWAS to avoid false positive findings.

This study has some limitations. Our analysis merely included individuals of European ancestry and the included population of the UKB study is young and health-oriented; thus, caution is needed when generalizing the results to other populations (45). Some of the included diseases in this study have a very low prevalence in the UKB; thus, some of the non-significant results may be due to a lack of power.

## Conclusion

In conclusion, this large-scale study provided a comprehensive understanding of the genetic architecture of nine serum amino acids. The genetic risk loci we identified have provided valuable insights into the underlying biology of these serum amino acids. Furthermore, this study supports a potential causal role of genetically elevated levels of serum valine and increased risk of T2D, and elevated levels of serum alanine and BCAAs with higher levels of blood lipids. These findings enhance our understanding of the genetic factors influencing amino acid metabolism and their potential implications for various health conditions.

## Supplementary Material

lqae046_Supplemental_Files

## Data Availability

All the relevant publications and dataset access points are listed in the reference section, including publicly available GWAS summary data. The codes for each section of the analysis are available as supplementary files. For FUMA analysis, the following website was used to generate graphs: https://fuma.ctglab.nl/.
