# Supplementary material for: Unravelling genetic architecture of circulatory amino acid levels, and their effect on risk of complex disorders
Source: NAR Genom Bioinform. 2024 May 6;6(2):lqae046. doi: 10.1093/nargab/lqae046 (PMC11071119; doi:10.1093/nargab/lqae046)
Supplement: lqae046_Supplemental_Files [file lqae046_supplemental_files.zip › Supp.Figures1-18.docx]

**
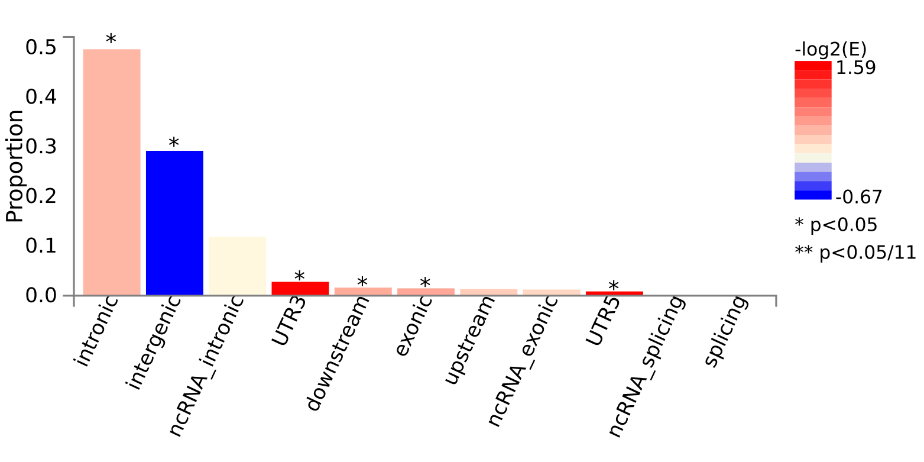
SuppFig1. Alanine**

**SuppFig2. Glutamine**


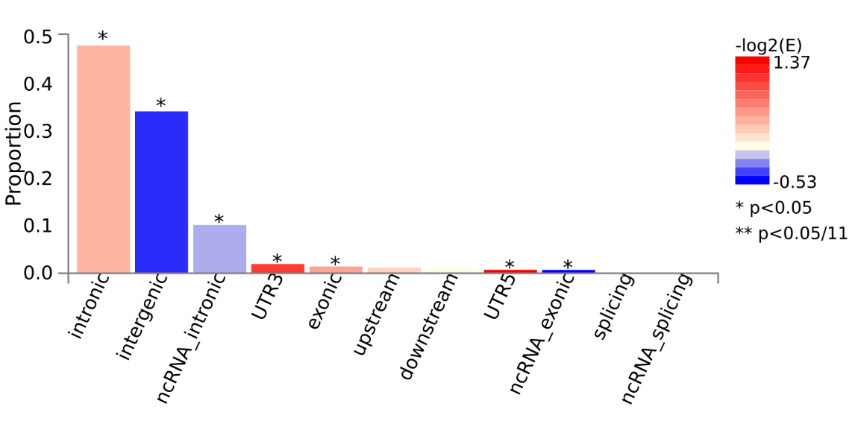


**
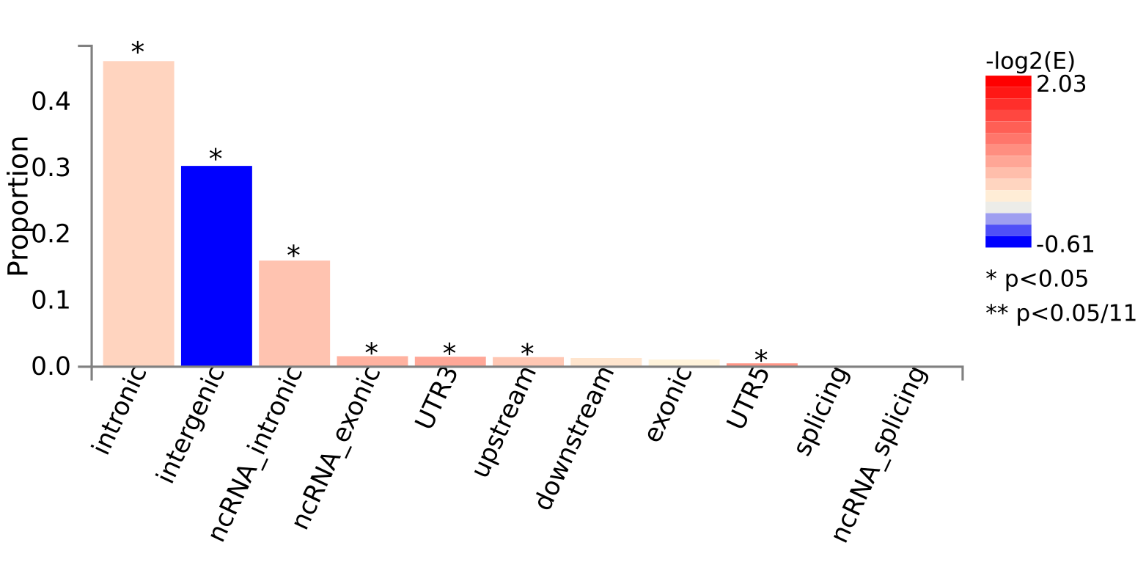
SuppFig3. Glycine**


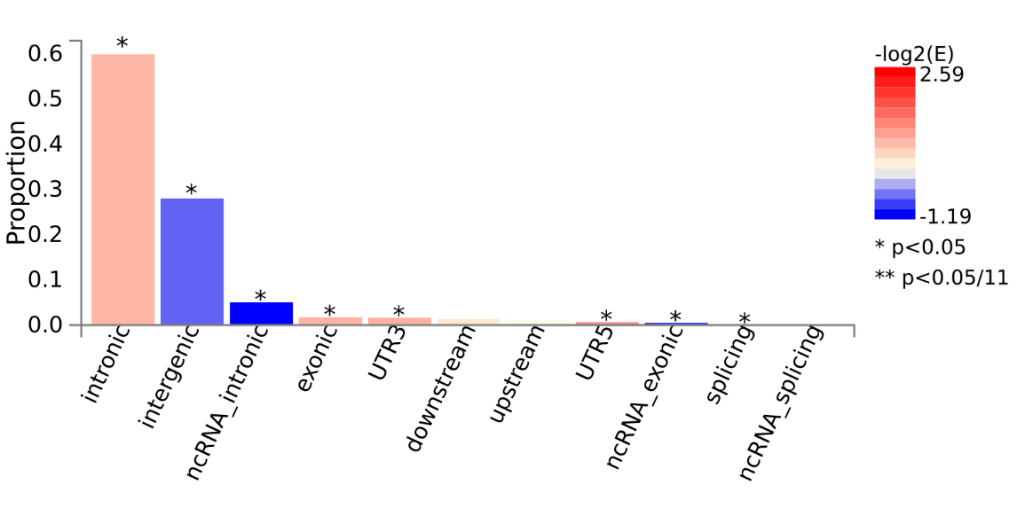
**SuppFig4. Histidine**


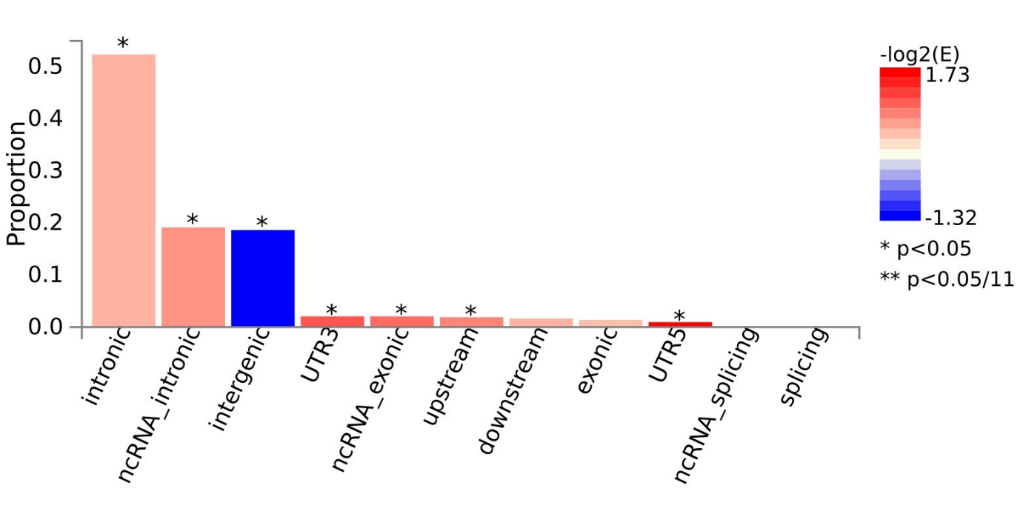
**SuppFig5. Isoleucine**


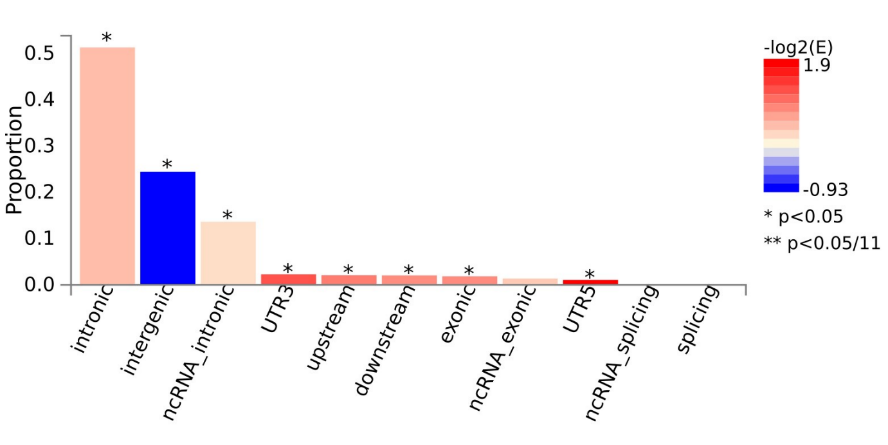
**SuppFig6.Leucine**


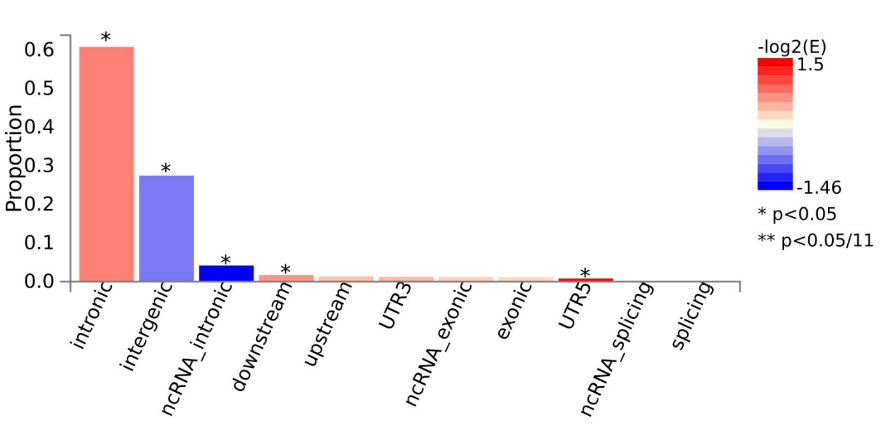
**SuppFig7.Phenylalanine**


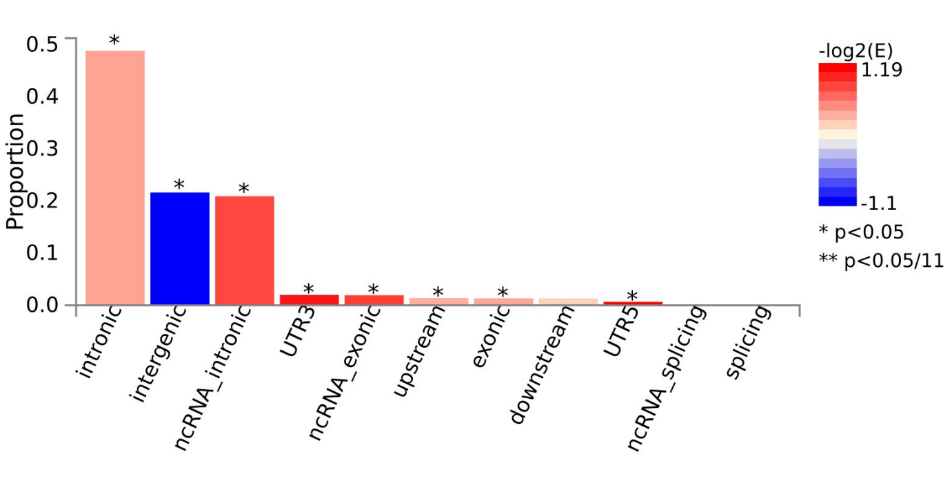
**SuppFig8.Tyrosine**


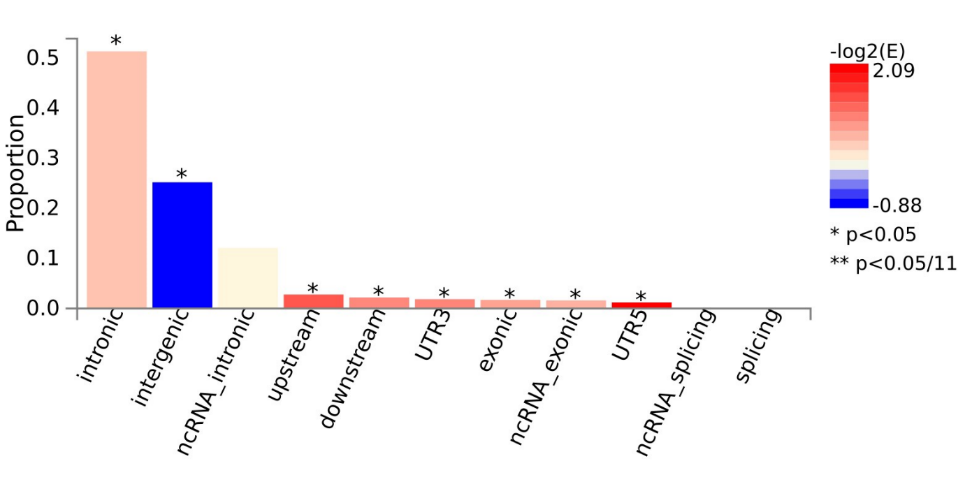
**SuppFig9.Valine**

**FigureS10. Gene-set analysis for serum alanine**


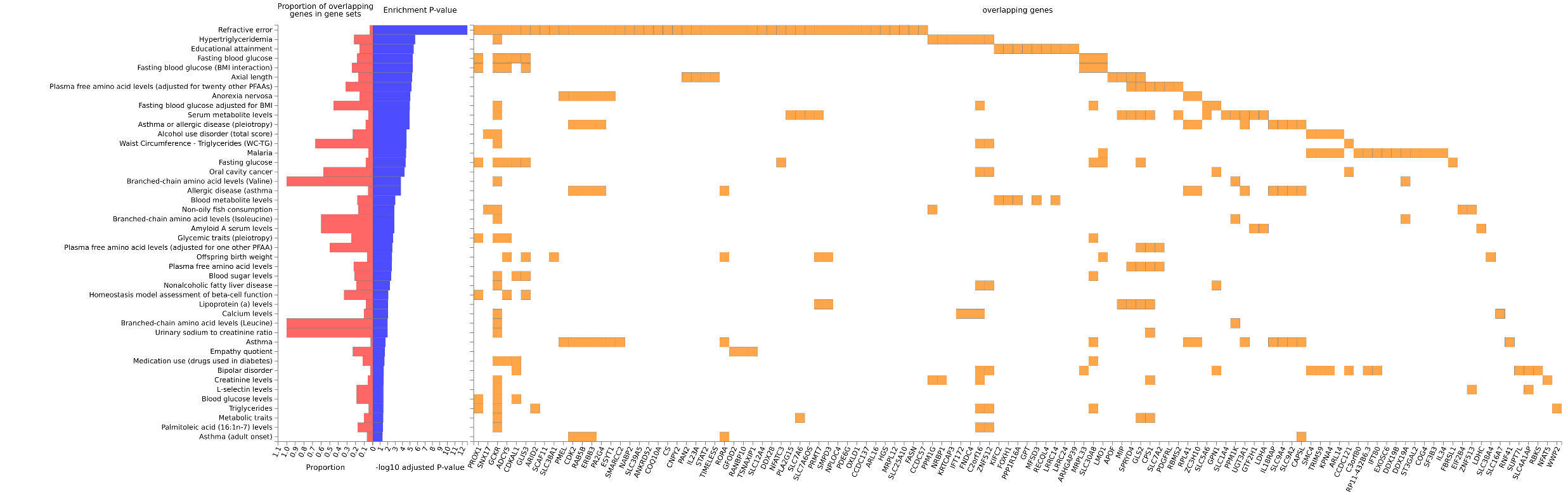


**FigureS11. Gene-set analysis for serum glycine**


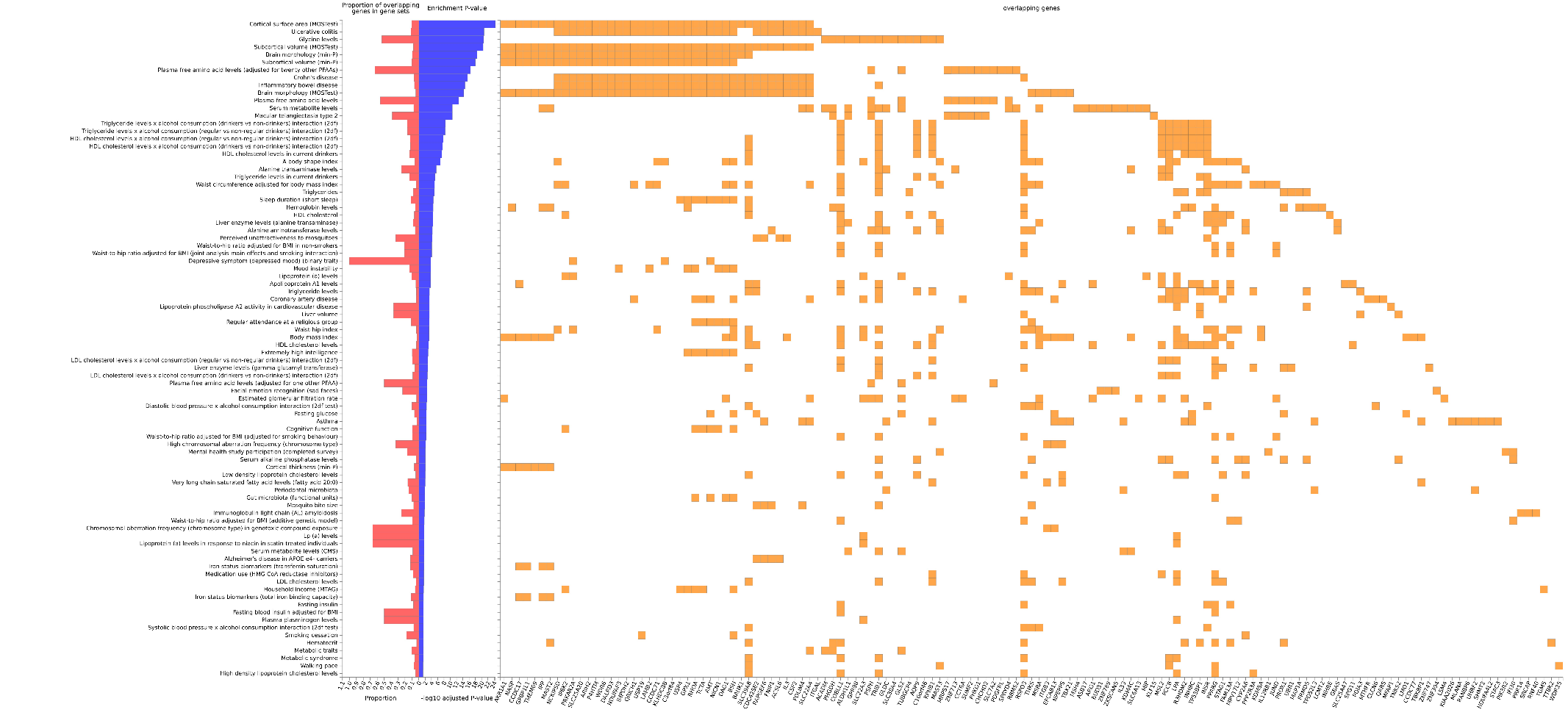


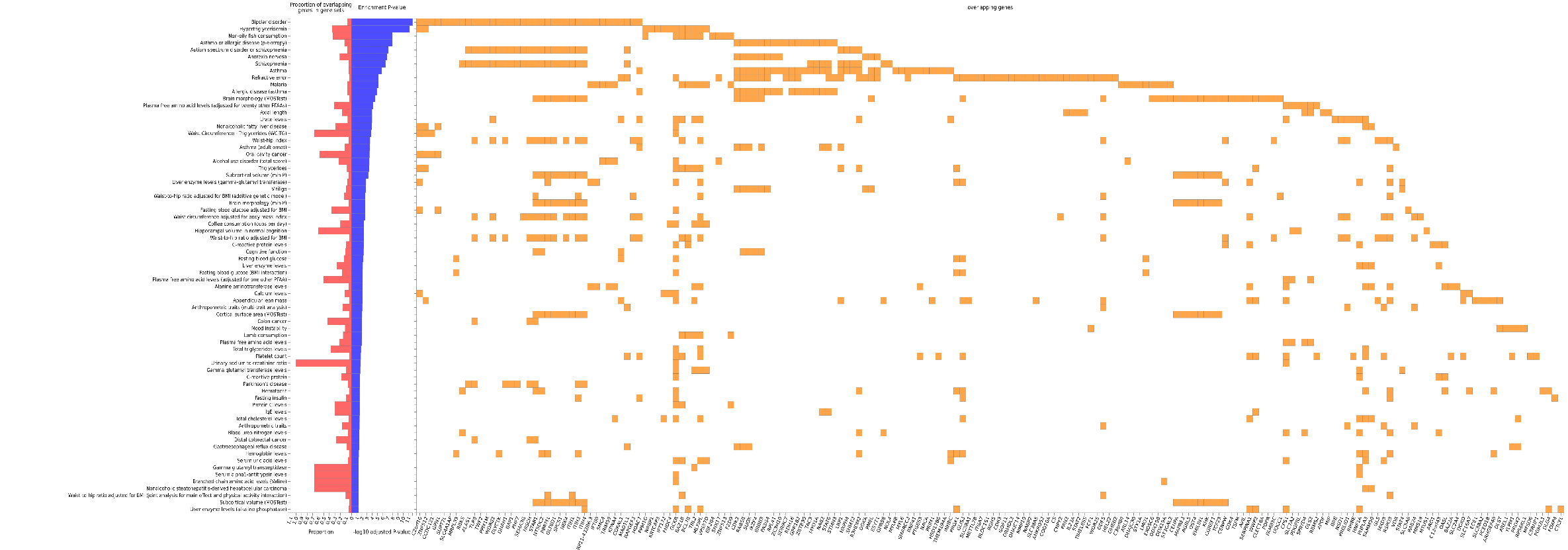
**FigureS12. Gene-set analysis for serum glutamine**

**FigureS13. Gene-set analysis for serum histidine**


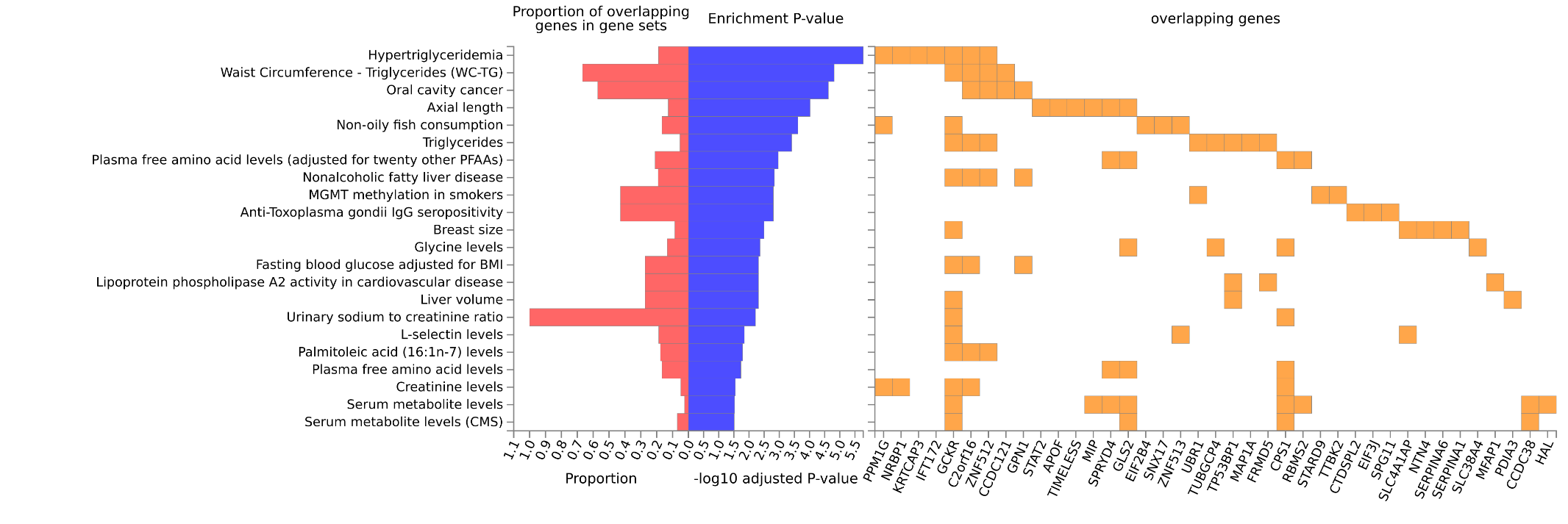


**FigureS14. Gene-set analysis for serum isoleucine**


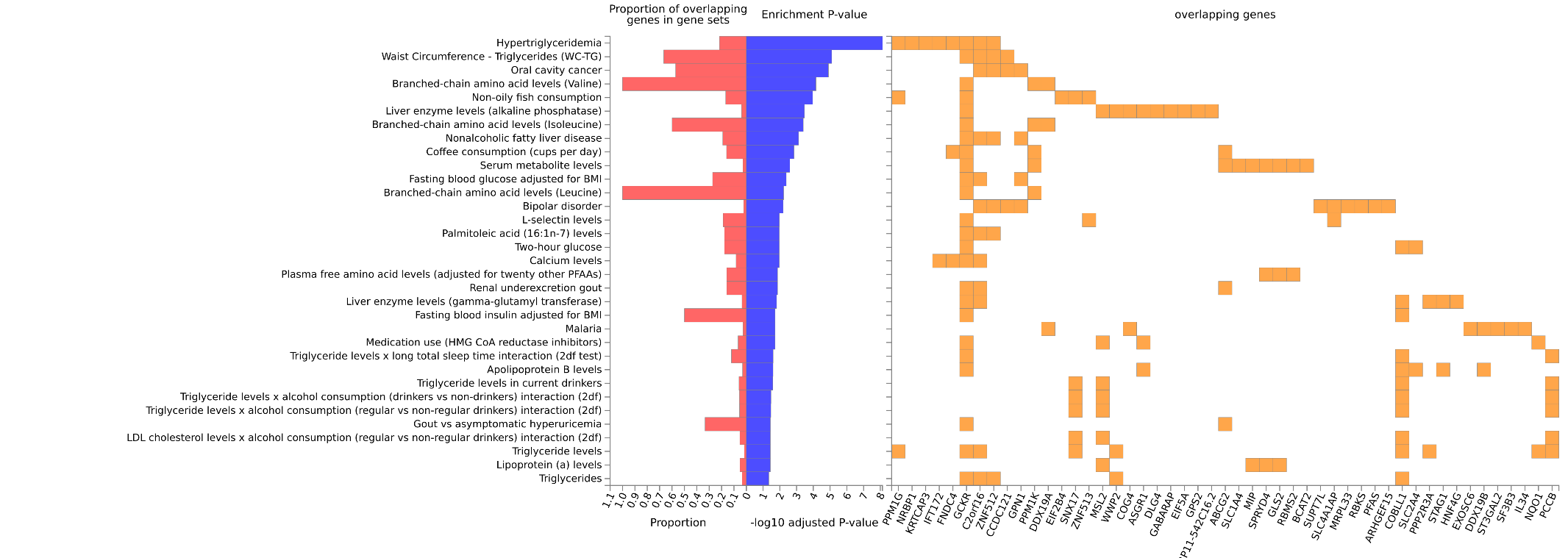


**FigureS15. Gene-set analysis for serum leucine**


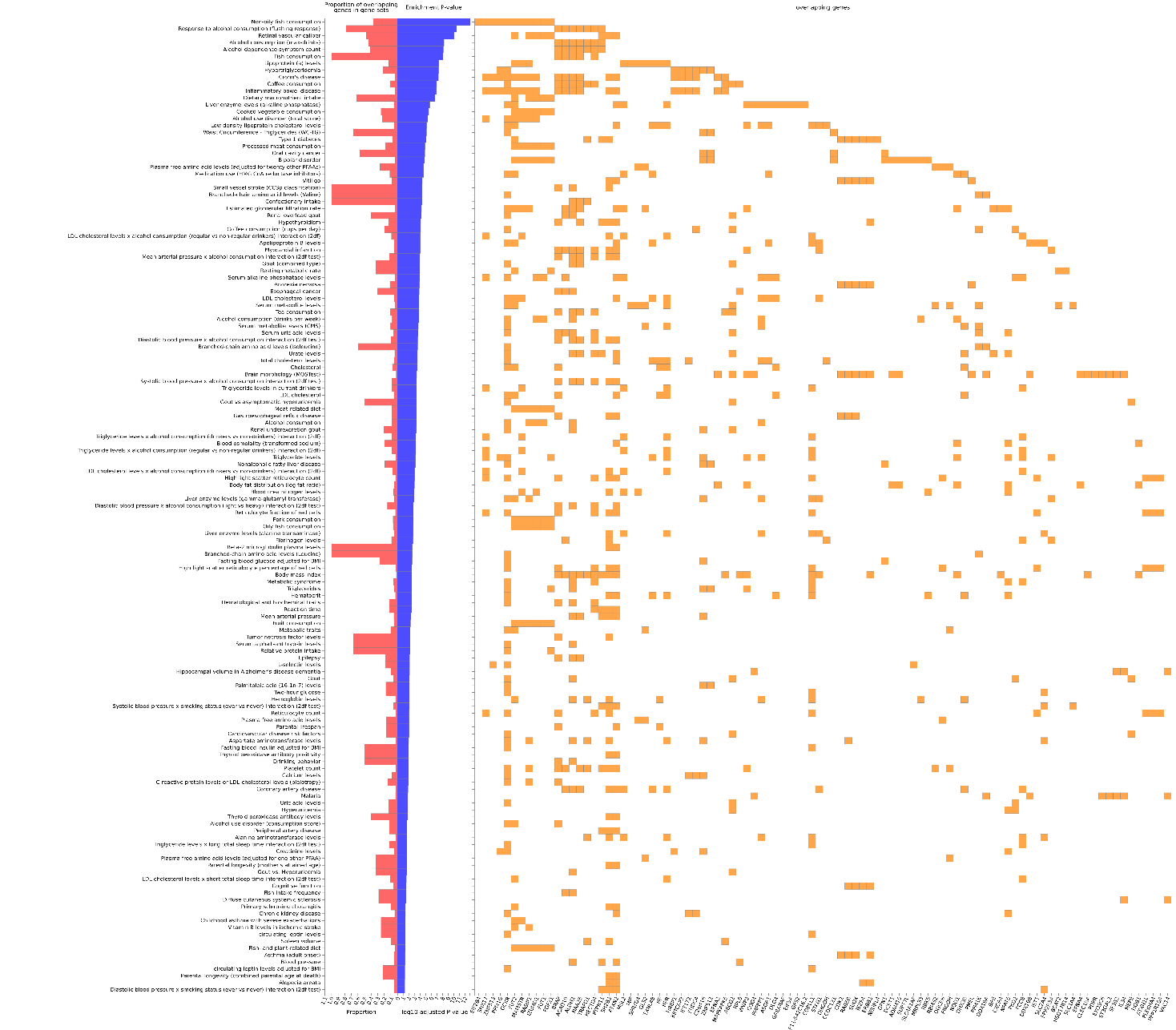


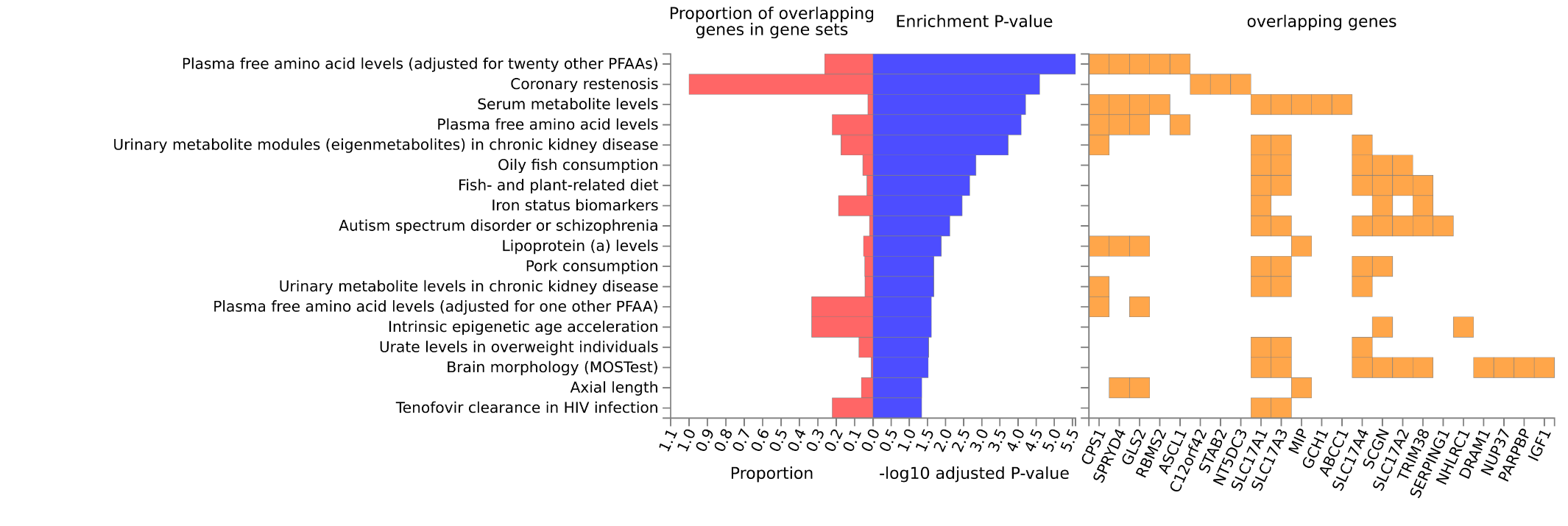
**FigureS16. Gene-set analysis for serum phenylalanine**

**
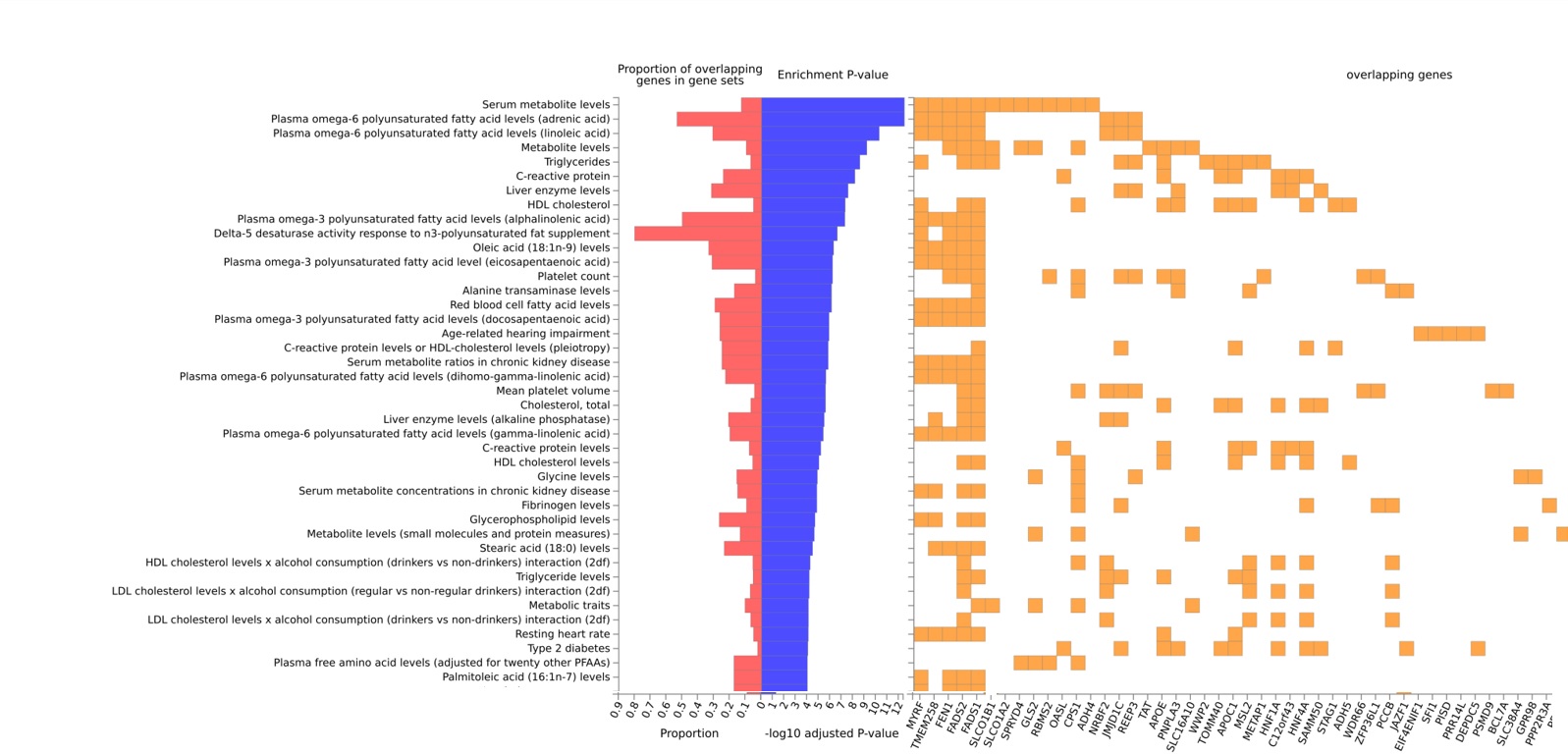
FigureS17. Gene-set analysis for serum tyrosine**


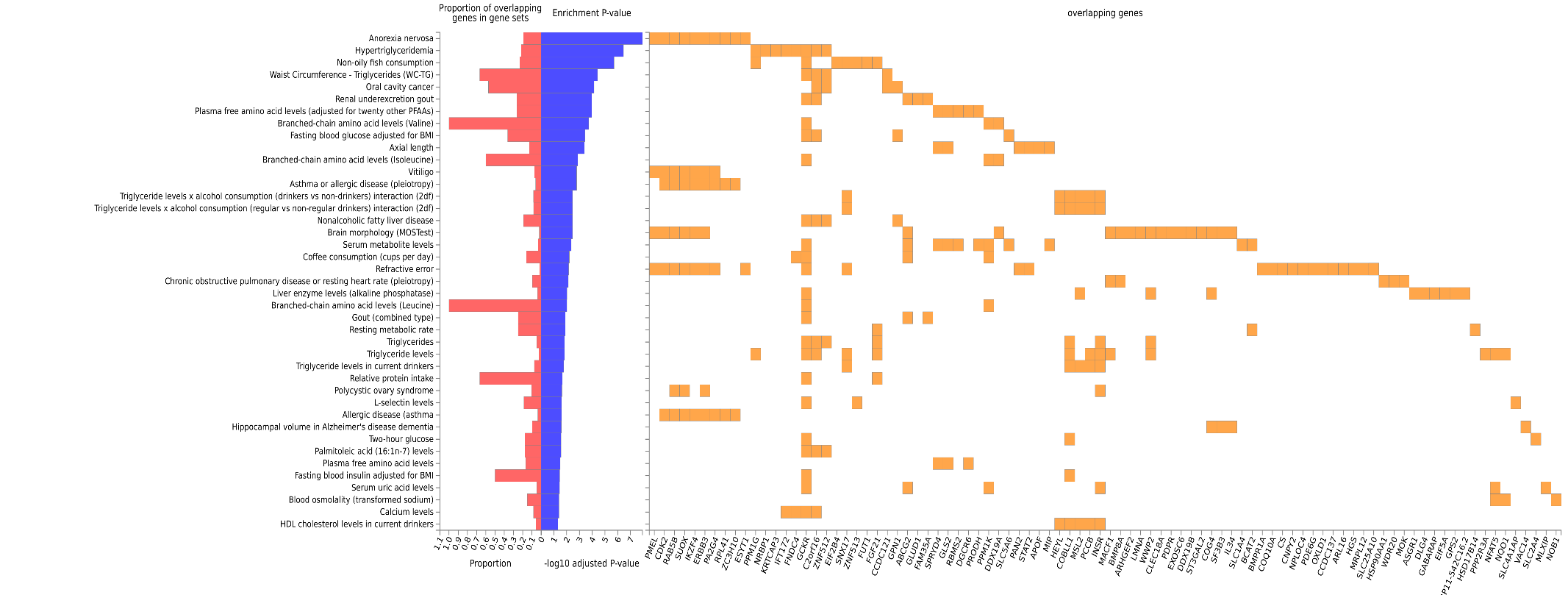
**FigureS18. Gene-set analysis for serum valine**
